# Postpartum Depression: Is Mode of Delivery a Risk Factor?

**DOI:** 10.5402/2012/616759

**Published:** 2012-12-13

**Authors:** Asli Goker, Emre Yanikkerem, M. Murat Demet, Serife Dikayak, Yasemin Yildirim, Faik M. Koyuncu

**Affiliations:** ^1^Department of Obstetrics and Gynecology, University of Celal Bayar, 45030 Manisa, Turkey; ^2^School of Nursing, University of Celal Bayar, 45030 Manisa, Turkey; ^3^Department of Psychiatry, University of Celal Bayar, 45030 Manisa, Turkey; ^4^Grand Medical Private Hospital, 45030 Manisa, Turkey

## Abstract

There are various factors related to postpartum depression. In this study we have aimed to determine the effect of mode of delivery on the risk of postpartum depression. A total of 318 women who applied for delivery were included in the study. Previously diagnosed fetal anomalies, preterm deliveries, stillbirths, and patients with need of intensive care unit were excluded from the study. Data about the patients were obtained during hospital stay. During the postpartum sixth week visit Edinburgh postnatal depression scale (EPDS) was applied. There was no significant difference between EPDS scores when compared according to age, education, gravidity, wanting the pregnancy, fear about birth, gender, family type, and income level (*P* > 0.05). Those who had experienced emesis during their pregnancy, had a history of depression, and were housewives had significantly higher EPDS scores (*P* < 0.05). Delivering by spontaneous vaginal birth, elective Cesarean section, or emergency Cesarean section had no effect on EPDS scores. In conclusion healthcare providers should be aware of postpartum depression risk in nonworking women with a history of emesis and depression and apply the EPDS to them for early detection of postpartum depression.

## 1. Introduction

Postpartum depression (PPD) is considered as an important health problem in modern societies. The prevalence of PND ranges from 7.6% to 39% in various areas of the world and differs according to the population tested and screening tools used [1–4]. The Diagnostic and Statistical Manual of Mental Disorders defines PPD as having five or more of the following symptoms for at least two weeks: insomnia/hypersomnia, psychomotor agitation or retardation, fatigue, appetite changes, feelings of hopelessness or guilt, decreased concentration, and suicidality. These episodes begin within 4 weeks postpartum and may last one year [5]. Risk factors that have been identified are poor marital relationship, prenatal depression, illness of the child, low socioeconomic status, low educational level, unwanted pregnancy, obesity, previous history of postpartum depression, and physical symptoms [6–10]. Some risk factors are merely seen in eastern communities such as sex of the infant [11, 12] and grand multiparity [13]. 

The importance of PPD lies in the fact that it is associated with long-term effects on family and child. Marital relationships are frequently affected [14]. Women with PPD tend to discontinue breastfeeding and cognitive development of the child is also shown to be impaired due to insufficient maternal-infant interaction [15, 16]. Therefore it is important to identify risk factors for PPD and to diagnose PPD in the early postpartum period to enable an immediate intervention. It is possible to screen for postpartum depression using the self-administered Edinburgh Postnatal Depression Scale (EPDS) [17] which is easy to apply and evaluate. Although it is not a diagnostic tool, a score above 13 is predictive of PPD and its sensitivity and specificity were found as 61.5% and 77.4%, respectively, in a Turkish validation study [18]. 

Complicated labour resulting in an emergency procedure has been identified as a potential risk factor for PPD in some studies but there are conflicting results showing no association with mode of delivery and risk of PPD [19, 20]. The rate of elective Cesarean section is rapidly increasing all over the world and it is important to identify whether the mode of delivery has an influence of maternal depression. Turkey's social structure is a mixture of modern Western European and traditional Anatolian but family bonds are tight even in urban areas. Women are usually supported by their families and friends during the puerperal period. During pregnancy and the puerperal period, women are followed up at primary health care centers on behalf of the Ministry of Health [21]. Doctors, nurses, and midwives working at these centers are the primary health professionals to contact puerperal women and identify PPD. This study aims to investigate the risk of postpartum depression after six weeks of delivery according to mode of delivery and to evaluate other related risk factors in order to develop precautions. 

## 2. Materials and Methods

The study was carried out at Celal Bayar University Faculty of Medicine Hospital, Department of Obstetrics and Gynecology, serving as a tertiary care facility. Approval of the local ethical committee and written informed consents from all participants were obtained. The inclusion criteria were delivery of a live baby at term. Exclusion criteria included multiple pregnancy, conception by assisted reproductive techniques, a previously diagnosed mental illness, antenatally diagnosed fetal anomaly, admission to the intensive care unit (either maternal or fetal), and being a single mother. 

Term delivery defined women who gave birth after the completed 37 weeks of gestational age. Patients were divided into three groups of vaginal delivery, planned Cesarean, and emergency Cesarean. A planned Cesarean was performed when a woman had elective Cesarean due to recurrent Cesarean, placenta previa, cephalopelvic disproportion, or presentation anomaly. An emergency Cesarean was performed to all kind of conditions threatening maternal or fetal life such as previously undiagnosed placental anomalies, fetal distress, preeclampsia, eclampsia, or fetal cord prolapse. 

On presentation to the hospital a form about sociodemographic parameters such as age, education, family type, education of husband, income level, working status, place of antenatal care was filled. Patient characteristics such as type of delivery, number of experienced pregnancies, births, and abortions, planning of current pregnancy, history of a medical condition during pregnancy, history of hyperemesis gravidarum and fear of delivery were recorded. 

At the sixth week postpartum visit patients were asked to fill out the Edinburgh Postpartum Depression Scale (EPDS). The EPDS is a 10-item self-administered questionnaire developed to screen depressive symptomatology over the past seven days in the postpartum period [17]. This is a reliable way of screening for postpartum depression with satisfactory specificity. EPDS consists of 10 items with 4 possible answers and sores from 0 to 3. The maximum score is 30. The validation of the Turkish version has been made by Aydin et al. [18]. A cut off value of 13 has been used to determine women at risk for depression. 

Descriptive data analysis for demographic variables were expressed as mean and standard deviation for continuous variables and number (percentage) for categorical variables. Statistical analysis was made using SPSS for windows version 15.0 (SPSS Inc., Chicago, IL, USA). Patient characteristics were analysed against EPDS scores and HAD scores by chi square test. Results were considered significant at *P* < 0.05. Subjects were categorised into two groups: EPDS scores ≥13 group at risk for depression and EPDS <12 group unlikely to be depressed. Categorical data were analysed using Pearson chi-square test and the differences between mean values of two groups were determined by parametric tests. 

## 3. Results 

This was a cross-sectional study. A total of 318 women had complete records and complete forms. There was no difference between groups of delivery mode in regard to sociodemographic variables. The mean age of the women was 27.74 ± 5.00 years ranging between 18 and 43. When categorised, most of the patients were under 35 (90.3%). Sociodemographic characteristics of the subjects are shown in Table 1. 

The majority of women were housewives (80.2%), lived in nuclear families (83.3%) and in the city (71.4%). The education level of women and husband was secondary education or less and high school or more in 63.8%, 36.2% and 59.4%, 40.6%, resp.). Income level was described as low or middle-high by 59.4% and 40.6%, respectively. Most of the patients were receiving antenatal care at the same hospital (58.5%), one third were sent from community hospitals due to medical indications (32.1%), and 9.4% had a private doctor. In 43 cases a complication during pregnancy was recorded (hypertensive disorder, gestational diabetes, thyroid disorder, urinary system infection, etc.). Only 5 of the women (1.6%) declared being unhappy with the baby's gender, of those 4 had a male baby. 

Mode of delivery was 33.0% for spontaneous vaginal, 33.6% for elective Cesarean, and 33.3% for emergency Cesarean. The indications for women who had a planned Cesarean section were: recurrent Cesarean (21.4%), cephalopelvic disproportion (6.0%), breech presentation (2.2%), and other (epilepsy, placenta previa, lumbar hernia, etc.). Emergency Cesarean was mostly performed for nonprogressive labor (8.5%), fetal distress (7.2%), recurrent Cesarean presenting with labour (6.6%), preeclampsia (2.8%) and other (cephalo pelvic disproportion, vulvar lesion, etc.). During the study period none of the women had delivered by instrumental delivery.

The mean score for EPDS was 9.4 ± 6.17 (min: 0, max: 28) among all women.  Table 2 demonstrates variables associated with postpartum depression at postpartum sixth week. A history of hyperemesis gravidarum, depression, and being a housewife were significantly associated with the risk of postpartum depression. The overall frequency of risk of depression was 31.4% among all the women. When calculated according to mode of delivery, it was found as 27.6%, 31.8%, 34.9% for vaginal, elective Cesarean, and emergency Cesarean, respectively, with no significant difference between them. The other variables of sociodemographic parameters did not show a significant effect on postpartum depression scores at sixth week. Being a first time mother had a significant effect on anxiety scores (*P* = 0.016) similarly did a history of depression (*P* = 0.005). 

## 4. Discussion

In this study the rate of PPD at six weeks postpartum (with a cut-off of ≥13 for the EPDS) was found as 31.4% among all the women. This result is relatively high when compared to those reported in three other Turkish studies conducted in Manisa, Izmir, and Erzurum, where the rates were 14.0%, 16.8%, and 14.0%, respectively [22–24], but similar to the study by Dindar and Erdogan where the cut-off score was 12 and the prevalence was reported as 25.6% [25]. These differences may be due to the variations in the application of the tests (postpartum one or six weeks, one year). There are different results on the prevalence of PPD ranging from 8.4% to 39% [2–4, 26] depending on the time of tests, sample size, design of the study (prospective versus retrospective), cut-off values of EPDS, and even type of test (EPDS; Beck depression inventory, etc.). 

There was no association between postpartum depression risk and mode of delivery but we found three other varibles affecting mood. These variables were history of depression, history of hyperemesis gravidarum, and being a housewife. Prior history of depression has been found to be an important factor in many studies independent of where it was done. [3, 4, 24, 25, 27] and our results are consistent. Women prone to depression should be categorised as high risk patients during and after pregnancy and family physicians as well as obstetricians or midwives should be aware of the increased risk of PPD in this group. Psychiatric consultation may be helpful during the peripartum or postpartum period.

 Complications during pregnancy, such as hyperemesis or premature contractions, were shown to be risk factors for PND [28]. We have also found that a history of hyperemesis gravidarum (HG) in the current pregnancy increases scores of EPDS. Another study from northeastern Turkey found history of emesis as a risk factor for PPD [29]. It is postulated that women who experience HG tend to be more depressive and a recent study shows that mood and anxiety disorders, and personality disturbances are frequently observed among women with HG [30]. Women diagnosed with HG during antenatal care should be followed closely after delivery and screening for PPD should be performed. 

Being a housewife increased the risk of postpartum depression nearly twofold. Other studies from Greece and Beirut show that PPD was more frequent among unemployed women [3, 4]. Returning to social life after childbirth and earning her own money seems to be protective against mood disturbances may it be in a western or eastern community. Being stuck at home with the baby is a depressive factor for women in this study. It is noteworthy that housewives are at risk of depression both during pregnancy and postpartum. Keeping in mind that 61.2% of women are housewives in Turkey, health professionals should be alert in this patient group and provide counselling during antenatal care and after birth [31]. 

There are conflicting results about the effect of age on PPD. A study from eastern Turkey [32] and the prospective cohort study by Sword et al. specify age less than 25 as an important risk factor for PPD [1] as well as a study by Lanes et al. [26]. Breese McCoy et al. however found no difference between age groups in regard to PPD [2]. In the present study there was no difference between age groups. The mean age of our participants was 27.7 and the mean age for Turkish women to be a first time mother is 22.3 [33]. McMahon et al. showed that older first time mothers were at risk for PPD. Age may play an important role when older aged women conceive by assisted reproduction but in our study we excluded these patients. The correlation between age and risk of PPD was found as very weak in our study group.

In our study PPD was not associated with level of education which differs from reports from Canada and Lebanon which indicate a relationship between low educational level and risk of PPD [3, 26]. Educational level may be directly associated with household income which in turn may affect the women's concerns about the costs of the infant. However in Turkey the primary care provider of the family is considered the husband and level of education is not necessarily related to household income. Additionally we have found no increase in PPD in women living in a low income family which also conflicts with the results of Lanes et al. and Tannous et al. [26, 34]. 

Unplanned pregnancies, number of births or abortions, hospitalisation during pregnancy, and fear about delivery were not associated with PPD in this study. The importance of the newborn's gender changes according to cultures. A study from Sweden showed no association between fetal gender and risk of postpartum depression in the mother 6 weeks or 6 months after delivery in fact birth of a baby boy increased the risk of postpartum blues within first 5 days [35]. Studies from China and India report a significant increase in PPD in case of a female baby [12, 20]. A study from eastern Turkey showed that even the number of daughters at home were important in developing PPD [32]. In Turkey the economic income in the eastern regions depends on agriculture and having a son is important to continue the family name. Western cities are more modern and industrialised. Manisa is a western city where gender discrimination is not as usual as the eastern parts of Turkey [25, 32] and in our study infant gender was not related to PPD. When the women were asked whether they were content with the baby's sex, 98.4% answered “Yes”. This result is comparable to studies from western societies [7]. 

We have found no increase in PPD in women living in nuclear families, villages, and who were followed up in community hospitals. Several studies have investigated the importance of marital relationships and family interactions but this was out of the scope of our study. 

In the present study we have found no difference between EPDS scores and mode of delivery. The risk for PPD was found as 31.4% among all women, 27.6%, 31.8%, 34.9% for vaginal, elective Cesarean and emergency Cesarean, respectively. There is no consensus on the effect of mode of delivery on postnatal mood or depression. In this study patients were not included if they delivered prematurely, were admitted to intensive care unit (mother or baby), had a diagnosed mental illness or antenatally diagnosed fetal anomaly, multiple pregnancy, conceived by assisted reproductive techniques in order to rule out definitive risk factors for PND and focus on the effect of mode of delivery. Josefsson et al. similarly reported that delivery mode was not effective on PPD [28]. 

Eisenach et al. showed that acute pain response during vaginal birth increased postpartum depression risk [36] and proposed a careful pain control during delivery. A study by Goecke et al. showed that emergency Cesarean lead to low control over delivery and women tended to be more depressive when compared to elective Cesarean where the timing of birth is set [37]. Mode of delivery was not independently associated with postpartum depression in a prospective cohort study by Sword et al. where young maternal age, maternal hospital admission, non-initiation of breastfeeding, urinary incontinence, multiparity, low social status, and low physical health were predictive variables [1]. Results by Durick et al. indicate little cause for concern about the quality of mother-infant interactions following Cesarean deliveries [38].

A study that found Cesarean to be a risk factor for PPD related the risk to younger age at childbirth [39]. Another study by Boyce and Todd showed that when compared with women having spontaneous vaginal or forceps deliveries, women having an emergency Cesarean section had more than six times the risk of developing postnatal depression at three months postpartum [40]. 

In Beirut, a significantly higher proportion of women who delivered vaginally was depressed after delivery compared with those who delivered by caesarean section (21% versus 7%). This was explained by the fact that in Lebanon the medical system is highly privatised, and women of higher socioeconomic status are more likely to receive caesarean sections and this group was less depressed [3]. 

Our results of no association between postpartum depression and mode of delivery is consistent with other recent studies [2, 41, 42]. Studies that found a relationship between PPD and Cesarean section are mostly conducted in the last two decades. Currently regional anaesthesia is widely used and women are involved in childbirth, immediate breastfeeding, and postoperative pain management after operative delivery and this helps them to bond to their child. Cesarean section is more acceptable with low complication rates compared to the past. 

Even though some studies reported increased risk of PPD after emergency Cesarean [19, 40], we did not find such a result. This may be due to Turkish women's attitude towards fate and not realising that an emergency delivery means risk for the baby. In this study we have excluded cases of perinatal death or patients with need for intensive care units for the baby and/or mother because these conditions are causes for depression. By excluding these factors we have tried to analyse the sole effect of emergency Cesarean and have found it as ineffective in creating risk for PPD. 

 There are some limitations to this study. Data were collected by a cross-sectional design, and therefore relations between different factors that may contribute to depression cannot be determined accurately. The EPDS is a screening test and needs diagnostic confirmation by structured or semistructured interview. Thus, it may not be possible to draw accurate conclusions. A prospective study including prebirth mood and longitudinal studies with diagnostic confirmation need to be carried out for this question. The overall prevalence of risk for postpartum depression can be evaluated by field research but we have included only patients who had access to be treated at a university hospital. 

## 5. Conclusions 

We observed that mode of delivery had no significant impact on the development of postnatal depression; however other factors may interact such as history of depression, hyperemesis gravidarum, and being a housewife. Women presenting with these characteristics should be paid special attention. 

## Figures and Tables

**Table 1:** Sociodemographic characteristics.

	Number (%)
Gravidity	
1	125 (39.3)
2	110 (34.6)
3	45 (14.2)
≥4	38 (11.9)
Parity	
1	146 (45.9)
2	121 (38.1)
3	30 (9.4)
≥4	21 (6.6)
Miscarriage	
0	255 (80.2)
≥1	63 (19.8)
Complication during pregnancy	
Yes	43 (13.5)
No	275 (86.5)
Infant gender	
Female	168 (52.8)
Male	150 (47.2)
Fear of delivery	
Yes	224 (70.4)
No	94 (29.6)
Planned pregnancy	
Yes	215 (67.6)
No	103 (32.4)
History of depression	
Yes	37 (11.6)
No	281 (88.4)
History of emesis	
Yes	125 (39.3)
No	193 (60.7)
Mode of delivery	
Vaginal	105 (33.0)
Elective Cesarean	197 (33.6)
Emergency Cesarean	106 (33.3)

**Table 2:** Factors associated with PPD at 6th postpartum week.

Related factors	OR	95% CI	*P*
History of emesis	1.690	0.717–0.002	0.036
History of depression	4.353	0.343–0.793	0.000
Being housewife	1.992	0.712–0.968	0.048
